# Editorial: Measuring and Analysing Social Determinants of Health in the Era of Big Data

**DOI:** 10.3389/fpubh.2022.902942

**Published:** 2022-05-27

**Authors:** Yi Guo, Jiang Bian, Fei Wang

**Affiliations:** ^1^Department of Health Outcomes and Biomedical Informatics, College of Medicine, University of Florida, Gainesville, FL, United States; ^2^Department of Population Health Sciences, Weill Cornell Medical College, New York, NY, United States

**Keywords:** electronic health records, natural language processing, environmental determinants, outcomes, intervention

A large and rapidly growing body of literature has provided convicting evidence on the significant role of social determinants of health (SDoH) in affecting human health, wellbeing, and quality of life (1). The World Health Organization defines SDoH as the non-medical factors that describe the conditions in which people are born, grow, live, work, and age (2). These factors include social and environmental circumstances such as education, income, housing, transportation, food access, diet, physical activity, discrimination, neighborhood safety, and many more. SDoH are one of the major contributors to the widespread health disparities and health inequities. It is estimated that SDoH are responsible for up to 40 percent of all preventable deaths in the United States (US), yet better medical care only accounts for a much smaller 10–15 percent (3). All evidence suggests that efforts to improve health need to shift from focusing on clinical factors to considering SDoH as key drivers of health outcomes.

Recognizing the importance of SDoH in shaping human health, a committee established by the US. Institute of Medicine (now the National Academy of Medicine) recommended ways of capturing 12 standardized measures from 11 SDoH domains in electronic health records (EHRs) in 2014 (4). Since then, healthcare systems began to explore ways to capture and integrate SDoH data within patients' EHRs. For example, Kaiser Permanente Northwest developed a set of EHR-based data collection tools to facilitate SDoH documentation using the International Classification of Diseases, Tenth Revision, Clinical Modification (ICD-10-CM) social diagnostic codes (Z codes) (5), which are intended to document patients' social, economic, occupational, and psychosocial circumstances. However, despite the increasing efforts to collect SDoH, they are infrequently captured in EHRs. Recent studies have shown that the Z codes are rarely used by clinicians in clinical documentations (6, 7).

Considering the importance of SDoH and the lack of SDoH data in EHRs, the editors proposed this Research Topic to provide a forum for cutting-edge research on the development and application of novel methods for measuring and analyzing SDoH in health outcomes research. For example, much information on SDoH is captured in unstructured EHR fields as free-text narratives. Yu et al. developed a natural language processing (NLP) pipeline that can extract 15 categories of SDoH from clinical narratives using a transformer-based model [i.e., Bidirectional Encoder Representations from Transformers (BERT)]. These SDoH included gender, race, ethnicity, smoking, employment, education, alcohol use, substance use, marital status, occupation, language, physical activity, transportation, financial constraint, and social cohesion. Using EHRs from a large health system in the United States, the authors obtained about 1.8 million clinical notes from over 10 thousand lung cancer patients and examined the frequencies of these SDoH in each category. Hatef et al. evaluated a text mining approach (i.e., pattern matching with regular expressions) in identifying phrases related to 5 categories of housing issues using EHRs from a large multispecialty medical group in the United States. Collaborating with SDoH experts, the research team reviewed existing literature and coding standards, and developed phrases addressing each housing issue and pattern-matching algorithms. Using data in 2.5 million clinical notes from 20 thousand patients, the authors found that, compared to manual annotation, the regular expression approach had a high level (> 94%) of precision at the phrase, note, and patient levels across different housing issues, although the recall level was relatively low.

Four articles in this Research Topic reported results from empirical analyses of the impact of SDoH in diverse research areas, including urbanization (Fang et al.), treatment adherence (Daabek et al.), liver cancer survival (Wu et al.), and happiness of rural residents (Xu and Ge). Another four review or opinion articles discussed the importance of collecting SDoH, identified areas of improvement, and proposed action plans, in the areas of sexual minority health (Wu et al.), patient segmentation (Rezaeiahari), physiatry (i.e., physical medicine and rehabilitation) (Conic et al.), and pediatric cancer (Reeves et al.). The remaining article simulated time-to-event data under various missing mechanisms (e.g., missing not at random) and assessed the performance of machine learning missing data imputation techniques based on the Cox proportional hazard model (Guo et al.). Given the poor documentation of SDoH in EHRs, methods for handling missing data are much needed in health outcomes research.

Overall, although SDoH are important factors driving health outcomes, they are poorly documented in EHRs. Even when SDoH are documented in EHRs, they are buried in unstructured clinical narratives and thus not readily accessible for downstream studies of health outcomes. As a result, current clinical research mainly studies the impacts of clinical factors (e.g., disease history, medical treatment) on health outcomes (e.g., prognosis, survival), ignoring the perhaps more important SDoH (e.g., financial constraint, housing issues) as contributing factors. Advances in research methods such as NLP provides new opportunities for identifying and capturing SDoH. However, what is really needed is targeted (and tailored) SDoH collection supported by EHR-based data collection tools, rather than using the non-specific Z codes. First of all, not all SDoH factors are equally important across the continuum of patient care or research areas. For example, insurance and geographic location (e.g., rural vs. urban residency) are more important for acute care settings, whereas social support and living conditions are more important for post-acute care and outpatient settings. A deep understanding of important SDoH in different phases of patient care is required for designing effective interventions that aim to improve health outcomes. Further, different representations or measures of the same SDoH factor are likely needed in different research areas. For example, physical activity can be measured using self-report survey instruments or wearable devices, depending on the desired level of data granularity. There needs to be clear guidelines, by type of disease and patient care, that outline which standardized SDoH measures to collect and how they should be integrated with patient EHRs.

Another more realistic solution to the lack of SDoH in EHRs is linking SDoH from other data sources. SDoH data are widely available in many local and national data sources such as Bureau of Labor Statistics (e.g., employment), Department of Education (e.g., literacy), Census Bureau (e.g., food insecurity, poverty), Bureau of Justice Statistics (e.g., Incarceration), Environmental Protection Agency (e.g., environmental factors) and many more. Linking these SDoH data longitudinally to patient EHR data at either the individual- or contextual level is the next essential step in advancing health outcomes research.

## Author Contributions

YG drafted the manuscript. JB and FW reviewed and revised the manuscript. All authors approved the final version of manuscript.

## Funding

YG and JB were funded in part by the National Institutes of Health (NIH), National Cancer Institute (NCI) grants 5R01CA246418-02, 3R01CA246418-02S1, 1R21CA245858-01A1, 3R21CA245858-01A1S1, and 1R21CA253394-01A1, NIH National Institute on Aging (NIA) grant 5R21AG068717-02, and Centers for Disease Control and Prevention (CDC) grant U18DP006512.

## Conflict of Interest

The authors declare that the research was conducted in the absence of any commercial or financial relationships that could be construed as a potential conflict of interest.

## Publisher's Note

All claims expressed in this article are solely those of the authors and do not necessarily represent those of their affiliated organizations, or those of the publisher, the editors and the reviewers. Any product that may be evaluated in this article, or claim that may be made by its manufacturer, is not guaranteed or endorsed by the publisher.
